# Preparation and Bioactivity Assessment of Chitosan-1-Acetic Acid-5-Flurouracil Conjugates as Cancer Prodrugs

**DOI:** 10.3390/molecules22111629

**Published:** 2017-11-08

**Authors:** Mohsin O. Mohammed, Kameran S. Hussain, Nadia Q. Haj

**Affiliations:** 1 Department of Chemistry, College of Science, Kirkuk University, Kirkuk 00964, Iraq; hajnadia@yahoo.co.uk; 2 Department of Chemistry, College of Nursing, Kirkuk University, Kirkuk 00964, Iraq; Kameran.hussain@yahoo.com

**Keywords:** chitosan, 5-fluorouracil, cancer, drug release, chitosan-1-acetic acid-fluorouracil, colonic cancer

## Abstract

5-fluorouracil (5-FU) is a specific anti-cancer agent that is generally used to treat gastrointestinal, colorectal, and breast cancer. In this work, chitosan (CS) was extracted from local fish scales using an established method. 5-FU was then converted to 1-acetic acid-5-fluorouracil (FUAC) and reacted with this CS to prepare chitosan-1-acetic acid-5-fluorouracil (CS-FUAC) conjugates as a colon-specific prodrug. All compounds were characterized by Proton nuclear magnetic resonance (^1^H-NMR), Fourier-transform infrared (FTIR), and UV-visible spectroscopy. The synthesized compound was subjected to a chemical stability study in phosphate buffer (0.2 M, pH 7.4) and in KCl/HCl buffer (0.2 M, pH 1.2) at different time intervals (0–240 min) and incubation at 37 °C. This revealed a significantly greater stability and a longer half-life for the CS-FUAC than for FUAC. Hemolytic activity results indicated a much lower toxicity for CS-FUAC than for 5-FU and supported consideration of CS-FUAC for further biological screening and application trials. The percentage of FUAC in the conjugates was determined by subjecting the prodrug to treatment in basic media to hydrolyze the amide bond, followed by absorbency measurements at 273 nm. The cytotoxicity studies of the conjugates were also evaluated on human colorectal cancer cell line (HT-29), which showed that the conjugates are more cytotoxic than the free drug. Therefore, CS-FUAC conjugates can be considered to represent potential colon-specific drug delivery agents, with minimal undesirable side effects, for colon cancer therapy.

## 1. Introduction

Cancer is the main cause of death throughout the world. In 2008, 7.6 million cancer deaths were recorded, which accounted for approximately 13% of all deaths in that year. Cancer occurs as different types, such as prostate, lung, colon, and breast cancer, but among these different types colon cancer causes the most deaths worldwide [1]. Colorectal cancer is treated with several drugs, including oxaliplatin, 5-fluorouracil (5-FU), folinic acid, methotrexate, bevacizumab (Avastin), and celecoxib (Celebrex). However, most of these drugs have some limitations, because they are ingested through the upper gastrointestinal tract (GIT) and they have a short half-life—characteristics that preclude an effective convergence of medications at the desired tumor site. Furthermore, some of these drugs have opposing side effects due to their nonspecific selectivity [2]. The utilization of customary oral plans, which are supposed to achieve conveyance of the chemotherapeutic agents to the tumor site, is restricted in colon cancer patients, since systemic conveyance results in the dissemination of the medication throughout the patient’s body and adds a greater risk of toxicity and dangerous side effects [3]. Specific conveyance of medications to the colon would provide the best restorative action by avoiding debasement or inactivation of the medication as it travels to the target site. All oral drug delivery strategies give rise to drug degradation, as the medications must transit through the stomach to reach the target site. This problem has been addressed by an assortment of methods for carrying drugs locally to the colon, including the use of timed-release capsules, pH subordinates, or enzyme dependent systems [4,5].

One specific anti-cancer agent generally used to treat gastrointestinal, colorectal, and breast cancer is the pyrimidine analog, 5-FU, which is used either alone or in a mixture with other anti-cancer drugs. Like other drugs, 5-FU has limitations, including a high rate of digestion in the body, a short half-life, an inclination to activate resistance in tumor cells, and cytotoxicity. These characteristics lead to a requirement for high doses of the drug, which increases the risk of side effects [6,7,8]. An ideal drug delivery system for 5-FU should therefore transfer the desired amount of the drug in small amounts and easily liberate it at the target site [8]. The available literature contains descriptions of a large number of drug delivery systems devised for 5-FU, including polymers, methacrylic-based and other nano gels, zeolites, poly (lactic-coglycolic acid), mesoporous silica, and chitosan (CS) [6,7,8,9,10,11,12,13,14].

**Figure 1:**
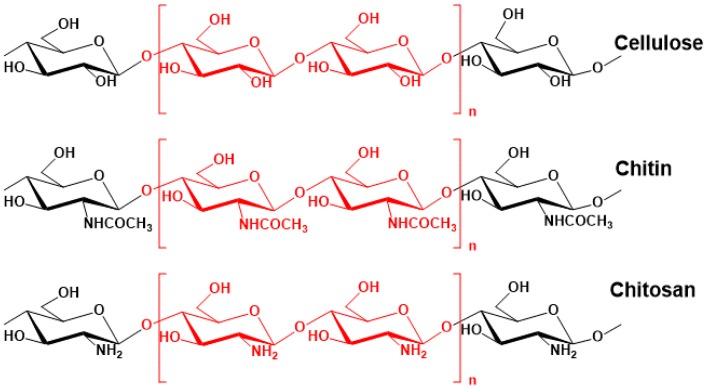
The chemical structures of chitin, chitosan and cellulose.

CS (Figure 1) is a polysaccharide containing repeated units of glucosamine and N-acetyl glucosamine units linked by β-(1,4)-glycosidic bonds. CS, as a fully or partly deacetylated form of chitin, is a very abundant natural resource. It has many applications due to its biodegradable and non-toxic properties and its biocompatibility [15]. These features also make CS a good candidate for use in the fabrication of prodrugs, which are medications bonded covalently to a carrier macromolecule (polymer). The goal of prodrug administration is to increase the level of the drug at the required location, such as a colon tumor target. Prodrugs typically enhance the drug’s physicochemical properties in order to minimize toxicity and unwanted side effects. 

A prodrug for colon cancer has several requirements: It should be nontoxic, biocompatible, and stable in the small intestine and stomach [16,17,18,19]. The present study describes the development of a colon-targeted delivery system for 5-FU prepared using a polymeric prodrug formulation. To achieve this, 5-FU was converted to 5-fluorouracil-1-acetic acid (FUAC) and loaded onto CS to prepare chitosan-1-acetic acid-5-fluorouracil (CS-FUAC) conjugates, which were then assessed for utilization in colon cancer therapy. The CS was extracted from fish scales collected from a local restaurant in Kirkuk City, Iraq, using an established chemical method. The product was characterized by Fourier-transform infrared and UV-spectrophotometry. This CS was loaded with 5-FU by linkage through an amide bond, and the resulting CS-FUAC product was tested for its potential as a 5-FU delivery agent.

## 2. Experimental

### 2.1. Materials and Methods

5-FU (≥99%), 1,1′-carbonyldiimidazole (CDI), sodium chlorite, potassium hydrogen carbonate (KHCO_3_), chloroacetic acid, sodium hydroxide, dimethylsulfoxide (DMSO), hydrochloric acid, and dialysis bags (*Mw* = 14,000) were purchased from Sigma-Aldrich Chemicals. Human colon carcinoma (HT-29) cell lines were collected from Kirkuk Health Directory (Kirkuk, Iraq) and cultured in Roswell Park Memorial Institute (RPMI 1640) medium supplemented with 1% penicillin-streptomycin and 10% Fetal bovine serum (FBS) under 5% CO_2_ atmosphere at 37 °C. The normal colonic fibroblast (CCD-18Co), catalogue number (CRL-1459), were purchased from the American Type Culture Collection cells and maintained in Dulbecco’s Modified Eagle’s Medium (DMEM) medium supplemented with 10% HI-FBS and 1% PS. Cells were cultured in a 5% CO_2_ in a humidified atmosphere at 37 °C. All chemicals used were of laboratory grade. FTIR spectra were recorded for the wave number range of 400–4000 cm^−1^ on a Perkin Elmer FTIR 4200 spectrometer under dry conditions at room temperature. ^1^H-NMR spectra were recorded using a Bruker Avance (500) spectrometer. UV-Visible analysis of the samples was carried out using a Perkin Elmer UV-Visible Spectrophotometer (Lamda-850) in the absorbance mode at wavelengths of 200–800 nm.

### 2.2. Extraction of Chitin and Chitosan from Fish Scales

Fish scales from the local fish market in Kirkuk City, Iraq were taken according to the published procedure [20]. Hydrogen chloride (HCL, 36.5 g/mol) 1% solution and Sodium hydroxide (NaOH, 40 g/mol) 0.5 N were prepared for demineralization and deproteinization receptivity.

### 2.3. Synthesis of CS-FUAC Conjugates

#### 2.3.1. Preparation of 1-Acetic acid-5-Fluorouracil (FUAC)

FUAC was prepared as published in the literature [21], with some modifications. An aqueous solution of KOH (0.60 g, 10.6 mmol, 10 mL) was added to 5-FU (1 g, 5.2 mmol). The reaction mixture was stirred at 100 °C for 80 min. Then a solution of chloroacetic acid (84 mL, 0.5 g, 5.2 mmol) was added gradually in an oil bath at 60 °C and stirred for 6 h. The result was acidified by adding diluted HCl to reach pH = 2 and cooled to 4 °C for 12 h. The precipitate formed was collected by filtration and redissolved in a saturated solution of KHCO_3_. Again, the solution was acidifying by diluted HCl to reach pH = 2 to get the needle-like crystals of FUAC (0.82 g, 60% yield).

#### 2.3.2. Preparation of CS-FUAC Conjugate

To a stirred solution of FUAC (0.376 g, 2 mmol) in 10 mL DMSO, (CDI) (0.652 g, 4 mmol) was added and the reaction mixture was stirred for 3 h to obtain 1-acetic acid-5-fluorouracilimidazoline. Then a solution of dissolved CS (0.264 g, 2 mmol) in aqueous glacial acetic acid (1%) was added. Triethylamine (TEA) 400 µL was added to the mixture and stirred at 60 °C for 20 h. The mixture was cooled and poured into a conical flask containing ethanol (100 mL). The precipitate was collected by filtration and washed with acetone 10 mL in a separation funnel to remove any remaining reactants and then the residue was dried in vacuum.

### 2.4. Chemical Structure Characterization

Structures of extracted CS, prepared FUAC and CS-FUAC conjugate were assessed by FT-IR spectroscopy. The ratio of 1:90 (*w*/*w*) from each compound and pre-dried KBr were mixed and compressed into a tablet. All the spectra were recorded in transmittance mode in the ranges of 4000–400 cm^−1^ with 32 scans at room temperature. Also, a UV spectrophotometer was used to analyze the compounds synthesized. This study was carried out at 273 nm. ^1^H- and ^13^C-NMR spectra was recorded on Bruker Avance (500) spectrometer in DMSO-d6 and 1% CF_3_COOD/D_2_O solution operating at 500 MHz at room temperature. 

### 2.5. The Percentage of Drug Content in the Conjugates 

The FUAC content in the conjugates was measured by the basic hydrolysis of the amide bond between the CS and FUAC. A series of standard solutions of FUAC in 1 N NaOH solution were prepared and a calibration curve (1–5 mg/mL) was plotted. The absorbency was calculated at 273 nm. A solution of CS-FUAC conjugates was prepared by dissolving the compound in 1 N NaOH (1 mg/mL), and their absorbance was also recorded at 273 nm. The percentage of FUAC in conjugates was measured by comparison with the standard. The following equation was used to calculate the percent of drug loading:

(1) $$\%\left(\frac{w}{w}\right)\;\mathrm{of}\;\mathrm{FUAC}\;\mathrm{loading}\;=\;\frac{\mathrm{FUAC}\;\mathrm{amount}}{\mathrm{CS}-\mathrm{FUAC}\;\mathrm{conjugates}\;\mathrm{amount}}\;\times 100$$

### 2.6. The In Vitro Drug Release Test

The response of the drug release from the conjugates was tested at 37 °C and the physiological pH 7.4 [22]. For every test, 20 mg of CS-FUAC and 4 mL Phosphate buffered saline (PBS) (Na_2_HPO_4_-KH_2_PO_4_) were mixed and the mixture was sealed in a dialysis bag. 200 mL PBS was added to a test tube and the dialysis bag was placed in and incubated in a water bath at 37 °C, 4 mL release medium was taken at selected time intervals for UV absorption analysis at a wavelength of 273 nm. Then a fresh in vitro medium was added after each sampling. The release analysis was achieved in triplicates for each sample and the average was acquiring. The percentage FUAC release (%, *w*/*w*) was examined as a function of release time. In order to study the half-life of CS-FUAC, UV spectra of the aqueous solutions of this compound and FUAC were carried out and their λ_max_ were recorded. According to the experimental conditions reported in [23], and since plotting log concentration against time resulted in a straight line, hydrolysis of the compound synthesized follows pseudo-first order kinetic and the experiential rate constant was measured from the slope of this plot. The degree of hydrolysis of the papered compounds were studied at different time intervals (0, 15, 30, 60,120 and 240 min); solution of (20 μg/mL) from the compound was taken in phosphate buffer (0.2 M, pH 7.4) and KCl/HCl buffer (0.2 M, pH 1.2) incubated at 37 °C. The half-life values were calculated from the pseudo-first order kinetic law.

### 2.7. In Vitro Cytotoxicity Study

The cytotoxicity study of the CS-FUAC conjugates and the free drug 5-FU against HT-29 cells was measured by MTT assay (3-(4,5-dimethylthiazol-2-yl)-2,5-diphenyltetrazolium bromide) [24]. A plate with 96-well was used and coated by approximately 4 × 10^3^ cells then different concentrations (1–10 M) of CS-FUAC conjugates and 5-FU were added for 12, 24 and 48 h. At the end of the time points, the media was removed from each well and refilled with fresh media (DMEM) supplemented with 10 µL of MTT (concentration-600 µg/mL). The cells were then incubated at 37 °C for 3–4 h. The resultant dark-blueformazan crystals were then solubilized in DMSO and the corresponding absorbance at 574 nm was recorded for each well by Cytation 3 multimode plate reader (Biotek, VT, USA). The acquired absorbance values were represented as percentage viable cells with respect to untreated control cells by the following equation:

(2) $$\mathrm{Relative}\;\mathrm{cell}\;\mathrm{viabillity}=\;\frac{\mathrm{Absorbance}\;\mathrm{of}\;\mathrm{the}\;\mathrm{sample}}{\;\mathrm{Absorbance}\;\mathrm{of}\;\mathrm{the}\;\mathrm{control}\;}\times 100$$

All experiments were carried out in triplicates and the results were plotted as mean ± SD.

### 2.8. Hemolytic Activity

The hemolytic study was carried out as the reported method for red blood cells (RBCs) suspension [25]. Blood from white rabbits was obtained and subjected to a centrifuge at 4000 rpm for 15 min, followed by the addition of a solution of normal saline (0.9% NaCl solution) to acquire the suspension (RBCs 2%). RBCs 2.0 mL dispersed in 8.0 mL normal saline solution as a negative control (making no hemolysis) and distilled water 8.0 mL was added to RBCs 2.0 mL as a positive control (making 100% hemolysis). A solution of FUAC and CS-FUAC concentration of 5, 1.8, 0.57, 0.2, 0.60 and 0.022 mmol/L, respectively, RBCs suspension, 2 mL and normal saline, 1 mL were incubated at 37 °C for 2 h, followed by centrifuge the result solution at 4000 rpm for 15 min. The supernatant was separated and the absorbance was calculated at 541 nm, the typical absorbance of hemoglobin (Hb) released from RBCs. Normal saline was used as a blank. The percentage of hemolysis was calculated by using the following equation:

(3) $$\%\;\mathrm{Hemolysis}=\;\frac{A\;\mathrm{sample}-A\;\mathrm{negative}\;\mathrm{control}}{A\;\mathrm{positive}\;\mathrm{control}-A\;\mathrm{negative}\;\mathrm{control}\;}\times 100$$

where, (A samples) are the UV-absorption of each compound at 541 nm, (A negative control) and (A positive control) raises to the negative control and positive control at 541 nm, respectively.

## 3. Results and Discussion

### 3.1. The Chemical Structure Characterization of CS

The schematic diagram for extraction of CS from fish scales is shown in (Figure S1, Supplementary Material). The FT-IR spectrum of the extracted CS (Figure S2, Supplementary Material) shows different stretching vibration bands as characteristic peaks in the range 3424–2883 cm^−1^ associated to ν (N–H) in ν (NH_2_) assoc. in amines [26]. The band range at 3424–3421 cm^−1^ may be related to ν (O–H), ν (N–H) and ν (NH_2_). The spectral bands between 1465–1040 cm^−1^ correspond to the stretching and bending vibrations of C–O, C–C and C–OH. Stretching vibration bands corresponding to the methyl group in NHCOCH_3_, methylene group in pyranose ring and a methylene group in CH_2_OH was showed in the range 2921–2879 cm^−1^. The effective of deacetylation was proved by which the band at 1597 cm^−1^ has a great intensity than at 1655 cm^−1^. The bands in the range 1605–1565 cm^−1^ corresponding to the primary amine groups, when the absorption bands at 3450, 3262, 3114 and 1658 cm^−1^ due to amide groups are missing from the deacetylated chitin and the absorption at 1590 cm^−1^ related to NH_2_ deformation of primary amines appears. The β-glucosidic linkages between the sugar units appeared as a sharp peak at 880 cm^−1^. An absorption band was observed in the range of 300–400 nm in the UV spectrum of CS at room temperature with 1 nm resolution as shown in Figure 3. The wavelength of CS was found to be 340 nm. This band absorption is related to the direct electronic d-π* orbitals and is called the Soret band.

### 3.2. Preparation and Characterization of CS-FUAC

**Scheme 1:**
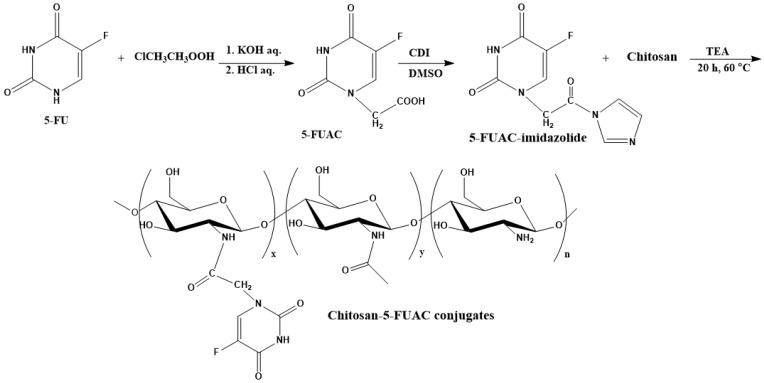
Preparation of chitosan-1-acetic acid-5-fluorouracil (CS-FUAC) conjugates.

**Figure 2:**
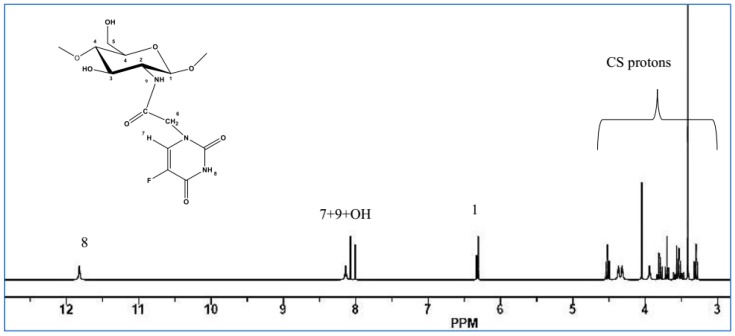
^1^H-NMR spectra of CS-FUAC.

**Figure 3:**
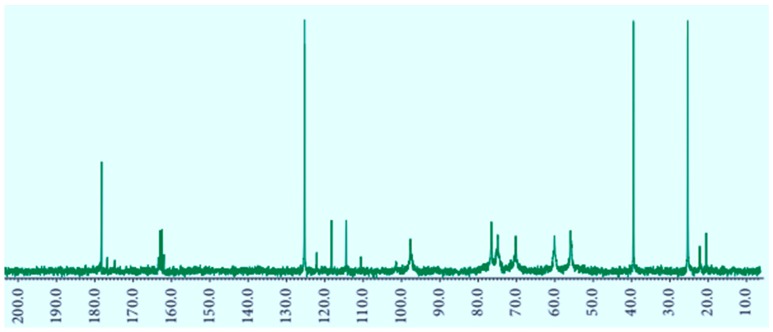
^13^C-NMR spectra of CS-FUAC.

**Figure 4:**
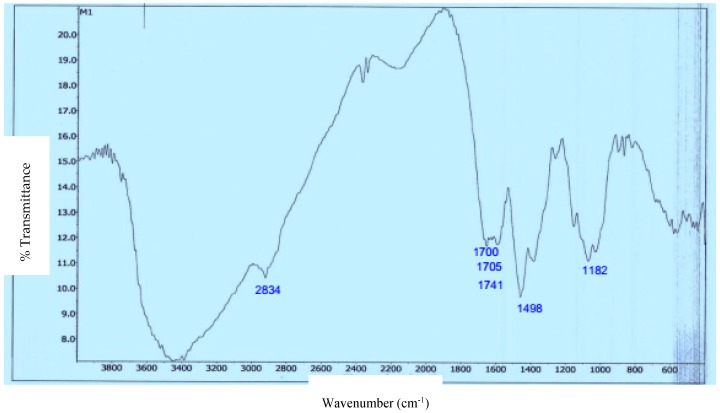
FT-IR spectra of CS-FUAC conjugate.

**Figure 5:**
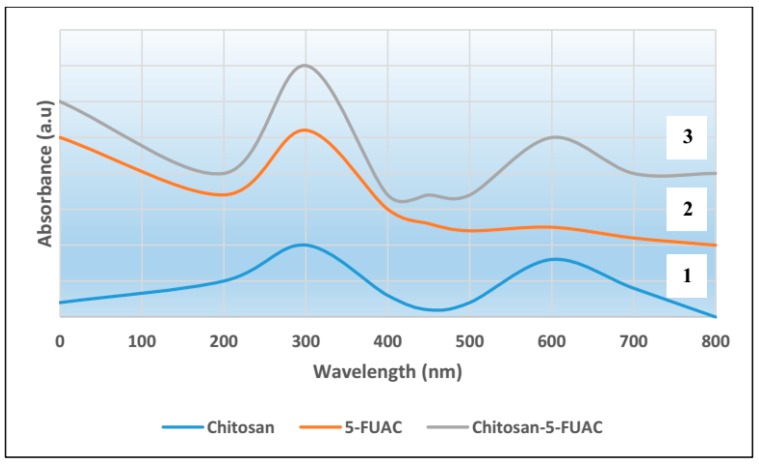
UV-vis spectra showing chitosan (CS) spectrum ‘1’, 1-acetic acid-5-fluorouracil (FUAC) spectrum ‘2’ and CS-FUAC conjugate spectrum ‘3’.

The preparation and the structure of CS-FUAC were confirmed by FTIR, ^1^H-NMR spectroscopy and UV-visible spectrophotometry. The CS-FUAC conjugate was prepared by the reaction of the imidazole derivative of FUAC with the extracted CS (Scheme 1). The process was started by converting 5-FU to the corresponding FUAC through an knowing method [21], treating the 5-FU with an aqueous solution of KOH and of chloroacetic acid in an oil bath at 60 °C for 6 h to yield a needle-like crystal of FUAC (0.82 g, 60% yield). The chemical structure and preparation of FUAC were proved by ^1^H-NMR and FT-IR Spectroscopy. The ^1^H-NMR spectra of FUAC (Figure S3, Supplementary Material), obviously shows the signals at δ 5.0 corresponding to the methylene of the carboxymethyl group and the signals at δ 7.4 and 8.0 ppm related to the NH and CH proton in the 5-FU ring. The FT-IR spectra of FUAC, indicates a characteristic peak at 3142 and 3062–2834 cm**^−^**^1^ are referring to the N–H, C–H stretching respectively. The Peaks at 1739, 1703 and 1658 cm**^−^**^1^ are corresponding to the C–O stretching in pyrimidine ring and COOH group respectively. The peaks due to the stretching mode of C–N, C–C, C–F and C–O appeared in a range of 1492–1179 cm**^−^**^1^. The FUAC-imidazole was synthesized in situ by homogeneous reaction of FUAC with imidazole in the presence of *N*,*N*-carbonyldiimidazole (CDI), then a solution of dissolved CS in aqueous acetic acid was added to the mixture in the presence of catalytic amount of triethylamine (TEA) [27]. The reaction was carried out using diverse mole ratios of CS: FUAC at the same temperature and time. The ^1^H-NMR spectra of CS-FUAC (Figure 2), obviously shows all the signals related to both CS and FUAC. Three signals at δ 4.3 corresponding to CH_2_ of the methylene group and the signals at δ 8.2 and 11.9 ppm due to the aromatic CH and NH proton of the 5-FU ring, confirm the successful coupling of 5-FUAC with CS. The chemical structure of the final compound was confirmed by ^13^C-NMR spectroscopy, Figure 3. Peaks at 175.02 and 23.05 ppm were found corresponding to C=O and CH_3_ in chitosan, respectively. Peaks at 26.25, 40.10 and 126.20 ppm were found due to the presence of carbon atoms in the FUAC moiety. The FT-IR spectrum of CS-FUAC conjugates (Figure 4) illustrated the characteristic peaks of both CS and FUAC. The FT-IR spectra of FUAC showed peaks at 3142 and 3062–2834 cm^−1^, corresponding to the N–H and C–H stretching respectively. Peaks related to C–O stretching of COOH group and C–O stretching in pyrimidine have appeared at 1660, 1741 and 1705 cm^−1^ respectively. The peaks between 1498–1182 cm^−1^ is corresponding to the C–N, C–C, C–F and C–O stretching mode respectively. The presence of a new peak at 1700 cm^−1^ confirms the formation of an amide bond between FUAC and CS. The chemical structures of CS, FUAC and CS-FUAC conjugates were proved by UV-vis spectroscopy Figure 5. As shown from the spectrum of CS-FUAC conjugate, a broad absorption band at 273 nm which indicates the presence of 5-FU in the conjugate. The characteristic absorption peak at 273 is due to the π → π* energy level transition of FUAC.

### 3.3. The percentage of Drug Content in CS-FUAC Conjugates

The percentage of FUAC conjugated to CS was measured by UV-vis spectroscopy. The experiment was started by hydrolysing the amide bond between the CS and FUAC in basic medium. As anticipated, by increasing the molar ratio of CS and FUAC the content of FUAC was increased. The FUAC content in the conjugates is shown in Table 1, when the molar ratios of CS and FUAC were 1:1 and 1:2 respectively.

### 3.4. The Chemical Stability of CS-FUAC Conjugates

**Figure 6:**
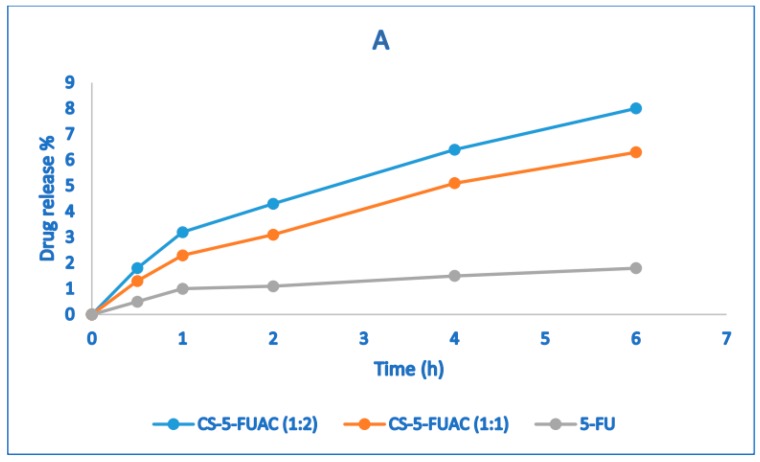

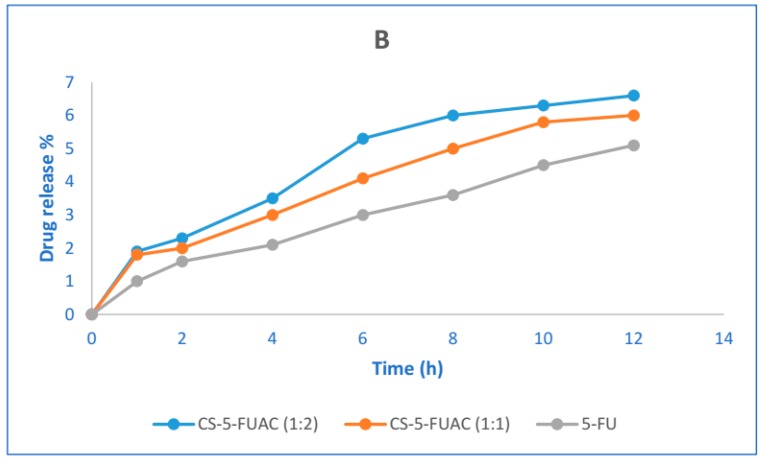
Stability test of CS-FUAC conjugates and free drug, 5-FU at (**A**) acidic pH 1.2, and (**B**) pH 7.4 buffer. All values are expressed as mean ± SD (n = 3).

The prodrug which targets the colon specifically should be stable at a wide range of pH during passing through the alimentary canal. In order to investigate the performance of the prepared prodrug in both acidic and basic conditions, two buffers of pH 1.2 and 7.4 were used to study the stability of the conjugate. Although the transition time is about 2 h in the stomach, the stability in pH 1.2 was carried out for 6 h (Figure 6A). Correspondingly, the stability of pH 7.4 was done for 12 h (Figure 6B), in spite of the fact that the transition time for small intestine is about 4 h. The result of the release patterns of the conjugates showed that just 3–5% release in acidic buffer was observed, while in the basic buffer it was 3–4%. The drug release in acidic conditions is higher when compared to the basic case and this may be because the acidic media works as a catalyst for the hydrolysis of an amide bond. The drug release of CS-FUAC is almost higher than the free drug 5-FU. In order to study the half-life of the conjugate, UV spectra of the aqueous solutions of the sodium salts of the CS-FUAC and FUAC were carried out and their λ_max_ were recorded. Since plotting log concentration against time resulted in a straight line and from the slope of this plot, the observed rate constant of hydrolysis was calculated, hydrolysis of the conjugate follows the pseudo-first order kinetic. The degree hydrolysis of the conjugate were carried out at different time intervals (0, 15, 30, 60, 120 and 240 min); solution of (20 μg/mL) from the conjugate was taken in phosphate buffer (0.2 M, pH 7.4) and KCl/HCl buffer (0.2M, pH 1.2), incubated at 37 °C for 240 min. The half-life values were calculated from the pseudo-first order kinetic law. The result showed that the conjugated compound has a t_1/2_ 3.48 in acidic media while t_1/2_ 15.26 in basic condition. Therefore, the above result has indicated significant increase in the values of t_1/2_ of CS-FUAC in comparison with FUAC at both acidic and slightly basic media pH 1.2 and pH 7.4 respectively [28]. 

### 3.5. In Vitro Cytotoxicity

**Figure 7:**
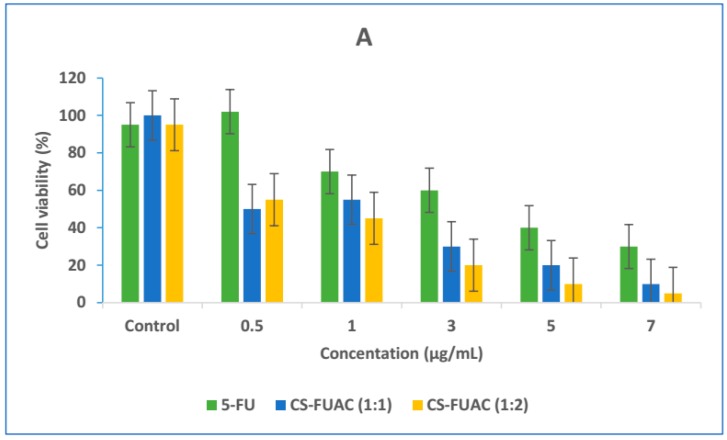

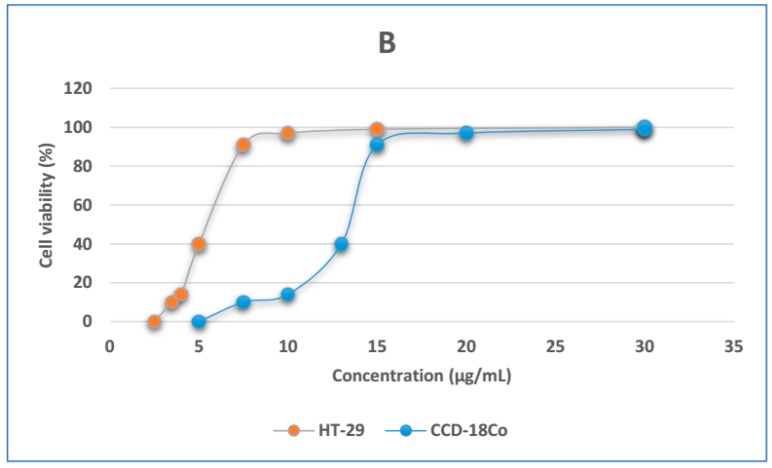
MTT viability assay of HT-29 cells treated with (0.5–7 μg/mL) of 5-FU and CS-FUAC conjugates, for 24 h (**A**); Dose response curves of the CS-5-FUAC on HT-29 and CCD-18Co cells in the concentration range 2.5–30 μg/mL (**B**). All values are expressed as mean ± SD (n = 3).

The HT-29 cell line was selected as a model of human colorectal adenocarcinoma cell line, and CCD-18Co human normal fibroblast was selected as a control cell line. The cell cytotoxicity of the CS-FUAC conjugates and 5-FU was tested against HT-29 (Figure 7A) at equal drug concentrations for 12, 24 and 48 h. Cell viability results obviously show that with increase in treatment time span and drug dosage levels there is subsequent increase in cytotoxicity of respective drug formulations. In addition, cytotoxicity assay also established that therapeutic efficacy of free drug 5-FU was drastically improved after conjugation with CS, which is probably an outcome of drug transition from a crystalline to an amorphous state. The IC_50_ of 5-FU against HT-29 cells (4.627 (g/mL) declined drastically to a value of 1.621 (g/mL) and 1.311 (g/mL) in case of CS-FUAC (1:1) and CS-FUAC (1:2) respectively. The second experiment was to test the cytotoxicity of the CS-FUAC on primary colon cell (Figure 7B). This study was showed that the papered compound was almost 2 times more cytotoxic on the colon cancer cells than on the normal cells, which indicates higher selectivity towards the colon cancer cells. The CCD-18Co normal cells, unlike HT-29 cells, were 2 folds less sensitive. In summary, it can be stated that, CS-FUAC conjugates synthesized in this work exhibits better cytotoxicity against colon cancer cells as compared to free drug as such. 

### 3.6. Hemolytic Activity

**Figure 8:**
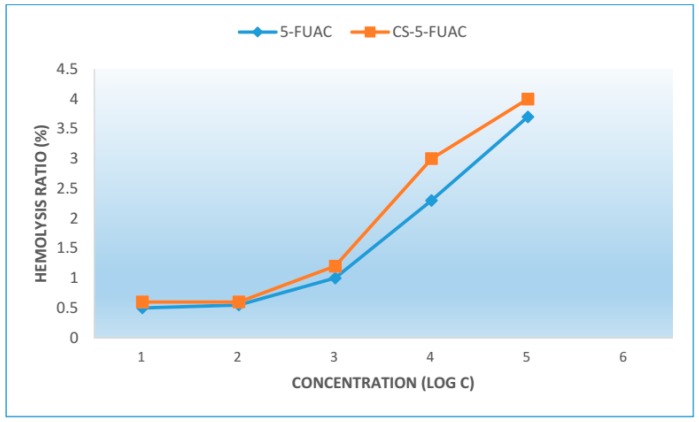
The percentage of red blood cell hemolysis incubated with CS-FUAC.

The result of hemolytic activity are shown in the Figure 8. The percentage of hemolysis caused by the CS-FUAC at different concentrations is studied and it can be observed that the percent hemolysis ratio increased with the increase of the concentration of CS-FUACs. All the CS-FUACs with a concentration above 660 μg/mL (Log *C* = −0.2 μg/mL) exhibit hemolysis below 5%.

## 4. Conclusions

In this research, CS-FUAC conjugates as probably colon-specific prodrugs were prepared using carbodiimide (CDI) as a chemical coupling agent. The conjugates prepared appeared to exhibit a higher stability in the basic condition. More significantly, conjugates prepared showed a higher half-life compared to the original drug. An in vitro cytotoxicity study clearly showed that these products are more active than the free drug. In Vitro cytotoxicity study clearly shows that these prodrugs conjugates are more active against human colorectal cancer cell lines (HT-29) compared to the free drug. Also, it was almost 2 times more cytotoxic on the colon cancer cells than on the normal cells. Based upon these results, it appears that CS-FUAC conjugates as prodrugs are potential candidates for colon-specific drug delivery. Further work to evaluate the biological effects of these molecules is under way.

## Figures and Tables

**Table 1:** Reaction data for CS-FUAC conjugates.

Sample ^i^	Drug loading (wt %) ^ii^	Yield (%)
CS-FUAC (1:1)	0.61	60
CS-FUAC (1:2)	0.75	68

^i^ All reaction was carried out at 75 °C for 24 h; ^ii^ Drug loading determined by UV.

## Supplementary Materials

Supplementary Material are available online. Supplementary data includes synthetic procedures and the 1H-NMR and IR, data for synthesised compounds associated with this article can be found, in the online version.
