# Supplementary material for: Preparation and Bioactivity Assessment of Chitosan-1-Acetic Acid-5-Flurouracil Conjugates as Cancer Prodrugs
Source: Molecules. 2017 Nov 8;22(11):1629. doi: 10.3390/molecules22111629 (PMC6150397; doi:10.3390/molecules22111629)
Supplement: Supplementary file 1 [file molecules-22-01629-s001.pdf]

Supporting information

Preparation and Bioactivity Assessment of Chitosan-1-Acetic Acid-5-Flurouracil Conjugates as Cancer Prodrugs

Mohsin Omar Mohammed[1], Kameran Shukur Hussein[2], Nadia Qader Haj[1]

[1]Department of Chemistry, College of Science, Kirkuk University, E-mail: Althker1@uokirkuk.edu.iq

[2]College of Nursing, Kirkuk University, E-mail: Kameran.hussain@yahoo.com

Experimental procedure for the production of Chitin and Chitosan

Preparation of chitosan from fish scales was performed using the general method comprising of demineralization, decolonization and deacetylation. Raw fish scales were washed thoroughly with water, dried in oven and soaked in 1% HCL solution for 36 hours. It was then washed dried in oven and kept in 2N NaOH solution for 36 hours for demineralization. Fish scales were then kept in potassium permanganate solution (having composition 1g of Oxalate acid in 100 mL water) for the process of decolourization of the experimental sample. These process resulted in chitin as the product which was further treated with 50% w/v NaOH for the process of deacetylation resulting in chitosan as the end product.[1]

Figure captions:

Figure S1 Extraction of Chitosan from fish scale.

Figure S2 FT-IR spectra of Chitosan.

Figure S3 ^{1}H NMR spectra of 1-acetic acid-5-Fluorouracil (FUAC).

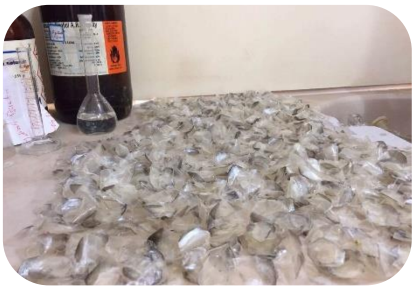

Fish scales

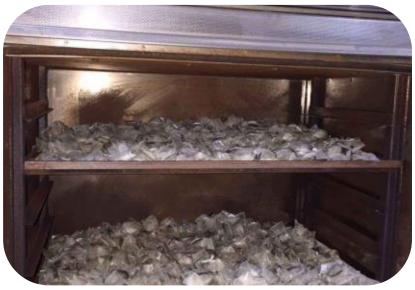

Washing and Drying

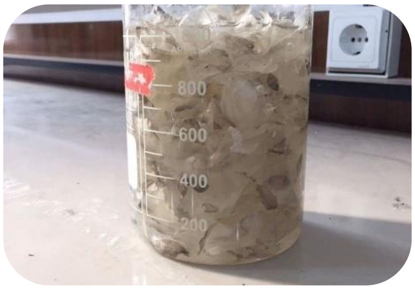

1% HCl

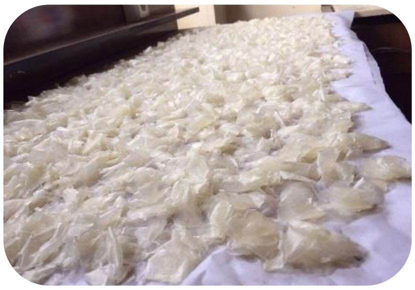

Drying

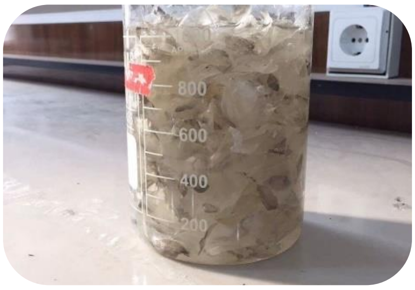

0.5 N NaOH

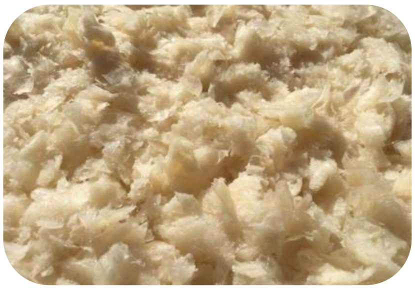

Drying

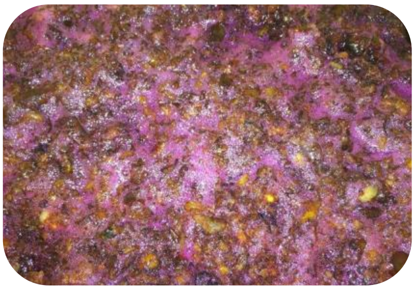

$KMnO_4$

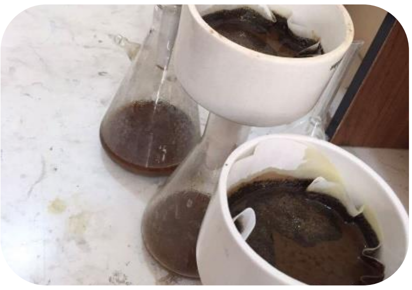

Filtring

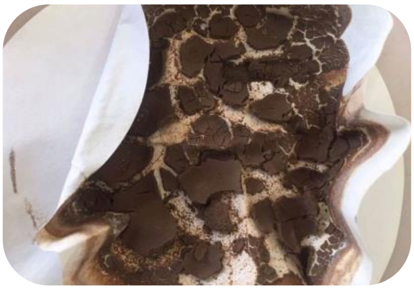

Chitosan

Figure S1 Extraction of Chitosan from fish scale

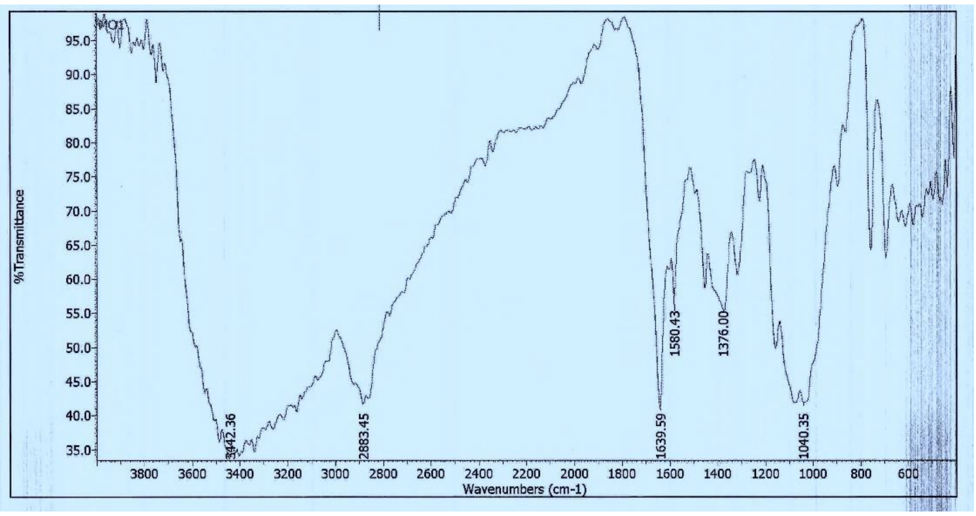

Figure S2 FT-IR spectra of Chitosan

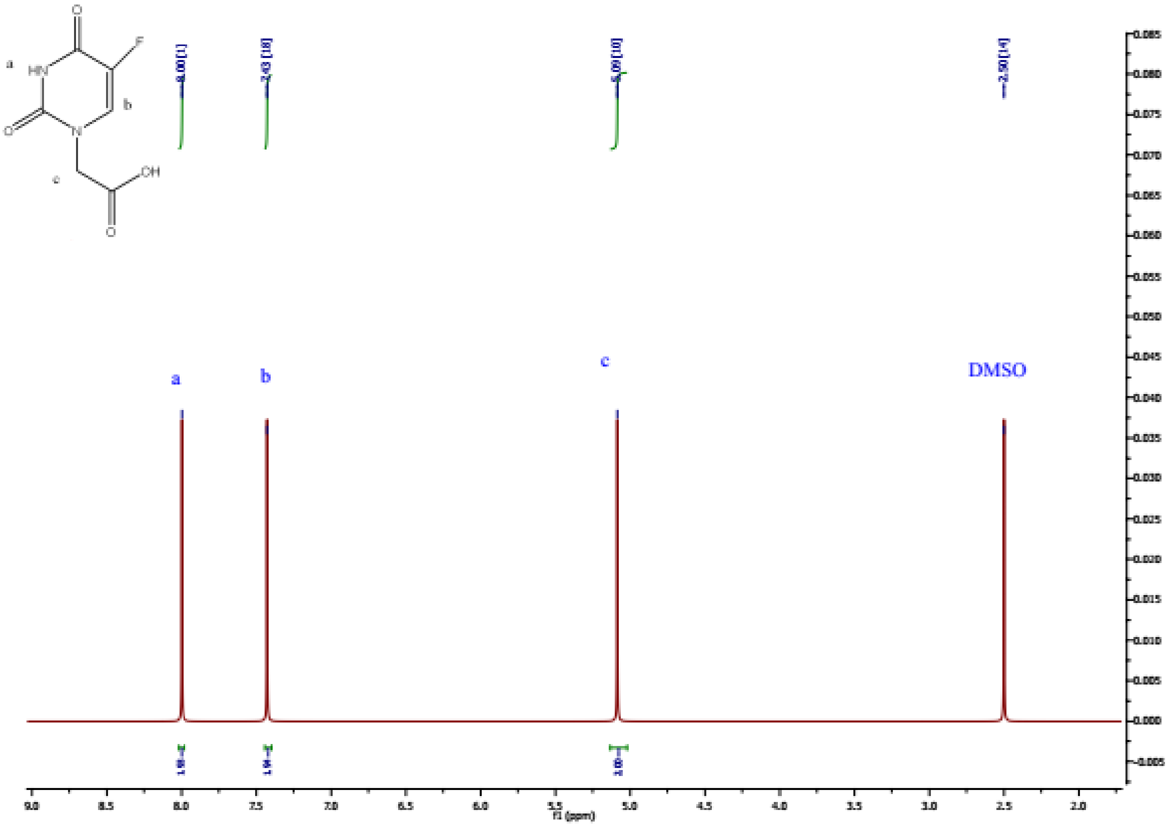

Figure S3 ^{1}H NMR spectra of 1-acetic acid-5-Fluorouracil (FUAC).
